# Supplementary material for: Structured thermal surface for radiative camouflage
Source: Nat Commun. 2018 Jan 18;9:273. doi: 10.1038/s41467-017-02678-8 (PMC5773602; doi:10.1038/s41467-017-02678-8)
Supplement: Supplementary file 1 — Supplementary Information [file 41467_2017_2678_MOESM1_ESM.pdf]

Supplementary Note 1

Thermal conductivity calculated with transformation thermotics

For the first transformation in equation (2) of the main article, the inverse is:

$$x = x', y = \delta[Ly' - (L - 2|x|)H]/(\delta L + (L - 2|x|)H). \tag{1}$$

The Jacobian J'can be calculated as:

$$J'(x',y') = \begin{bmatrix} 1 & 0 \\ G(x',y') & F(x') \end{bmatrix}, \tag{2}$$

Where

$$F(x') = \frac{(H+\delta)L-2H|x'|}{\delta L}, G(x',y') = -\frac{2(y'+\delta)H}{(H+\delta)L-2H|x'|}\frac{x'}{|x'|}. \tag{3}$$

The determinant is:

$$det J' = F(x'). \tag{4}$$

The anisotropic thermal conductivity κ' is then:

$$\kappa'(x',y') = \begin{bmatrix} 1 & G(x',y') \\ G(x',y') & G(x',y')^2 + F(x')^2 \end{bmatrix} \frac{\kappa_0}{F(x')}. \tag{5}$$

For the second transformation in the main article, it can be written as:

$$x'' = \begin{cases} \frac{a}{b}x' - \frac{b-a}{h}(2h - y') & \text{(Region A}_1\text{)} \\ \frac{a}{b}x' + \frac{b-a}{h}(2h - y') & \text{(Region A}_2\text{)} \\ \frac{a}{b}x' - \frac{b-a}{h}y' & \text{(Region A}_3\text{)} \\ \frac{a}{b}x' + \frac{b-a}{h}y' & \text{(Region A}_4\text{)} \end{cases}, y'' = y', \tag{6}$$

the inverse is:

$$x' = \frac{x''}{\alpha}\left[1 - \frac{\beta(h-|y''-h|)}{|x''|}\right], y' = y'', \tag{7}$$

where $\alpha = a/b$, $\beta = (b-a)/h$ $\alpha = a/b$, $\beta = (b - a)/h$. The Jacobian J''can be calculated as:

$$J_1'' = J_4'' = \begin{bmatrix} \alpha & \beta \\ 0 & 1 \end{bmatrix}, J_2'' = J_3'' = \begin{bmatrix} \alpha & -\beta \\ 0 & 1 \end{bmatrix} \tag{8}$$

and we have

$$det J_1'' = det J_2'' = det J_3'' = det J_4'' = \alpha \tag{9}$$

The anisotropic thermal conductivity κ'' is then:

$$\kappa''_B(x'',y'') = \begin{bmatrix} (\alpha+\beta G)^2+(\beta F)^2 & \alpha G+\beta(G^2+F^2) \\ \alpha G+\beta(G^2+F^2) & G^2+F^2 \end{bmatrix} \frac{\kappa_0}{\alpha F}, \tag{10}$$

$$\kappa''_C(x'',y'') = \begin{bmatrix} (\alpha-\beta G)^2+(\beta F)^2 & \alpha G-\beta(G^2+F^2) \\ \alpha G-\beta(G^2+F^2) & G^2+F^2 \end{bmatrix} \frac{\kappa_0}{\alpha F}, \tag{11}$$

where F and G are now functions of x'' and y'':

$$F(x'',y'') = F[x'(x'',y'')], G(x'',y'') = G[x'(x'',y''), y'(y'')]. \tag{12}$$

Also in Region A we have $\kappa''_{\mathrm{A}}(x'',y'') = \kappa'(x'',y'')$. The anisotropic thermal conductivity distribution is plotted in Supplementary Figure 1, with κ_0 = 20 W m^{-1} K^{-1} and δ= 2 mm.

Supplementary Note 2

Robustness against different background materials

We examine the performance of our structure for background materials with different thermal conductivities by calculating the curve of ΔT as defined in the main article. In Supplementary Figure 2b-d we plot the curves for the background with an object (Supplementary Figure 2b) and with the device (Supplementary Figure 2c and d. The curves for the device built with anisotropic material is not shown since the patterns are similar). It is evident that for a large range of background thermal conductivities κ_0, the device works properly. Of course, the performance cannot be maintained for arbitrarily small κ_0 because of the limitation of available materials as the insulating sub-layers of the structure. When κ_0 approaches the thermal conductivity of this sub-layer, it is not a very good approximation to take one component of the anisotropic thermal conductivity of the device to be zero. Therefore, we see some discrepancy in Supplementary Figure 2d with κ_0 lower than 1 W m^{-1} K^{-1}. However, even with such small background thermal conductivity, ΔT is still confined in ±0.7 K with a smooth profile. Detecting the device is still much harder than detecting a bare object with sharp temperature profile as in Supplementary Figure 2f-h.

Here we add another model, as schematically plotted in Supplementary Figure 2a, for comparison to show the necessity of the anisotropic structure. The model represents the strategy of covering the object with just a copper film (1 mm thick) and filling the space between the film and the object with PDMS. As can be seen from Supplementary Figure 2e, this strategy has only a limited effect (with a maximum deviation of 0.7 K, which is close to the performance in Supplementary Figure 2d with κ_0 = 0.5 W m^{-1} K^{-1}) for the background with high thermal conductivity (400 W m^{-1} K^{-1}). For other kinds of background the performance is even worse (note the range of ΔT in Supplementary Figure 2e is twice larger than those in Supplementary Figure 2b-d). Also, the profiles of ΔT with sharp peaks are easily detectable. The reason is that the isotropic structure will inevitably deform the isothermal lines due to the convex upper boundary, as shown in Supplementary Figure 2f-h.

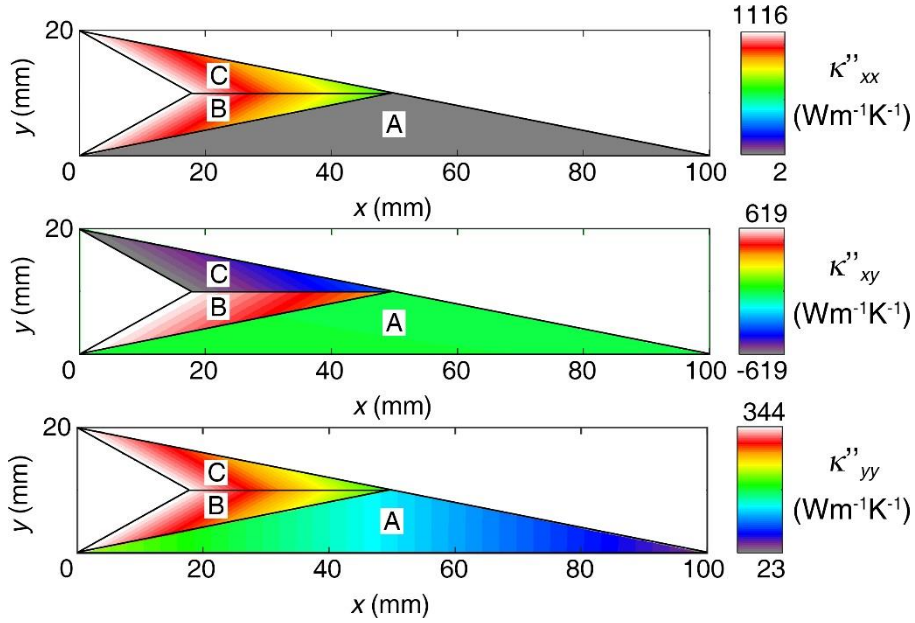

Supplementary Figure 1. The thermal conductivity tensor κ'' profile of the device. Because of symmetry only the $x > 0$ parts are plotted. The components κ''_{xx}, κ''_{xy} and κ''_{yy} of the tensor in Regions A, B and C are calculated according to the Supplementary Equation (5), (10) and (11) respectively, with κ_0 = 20 W m^{-1} K^{-1} and δ = 2 mm.

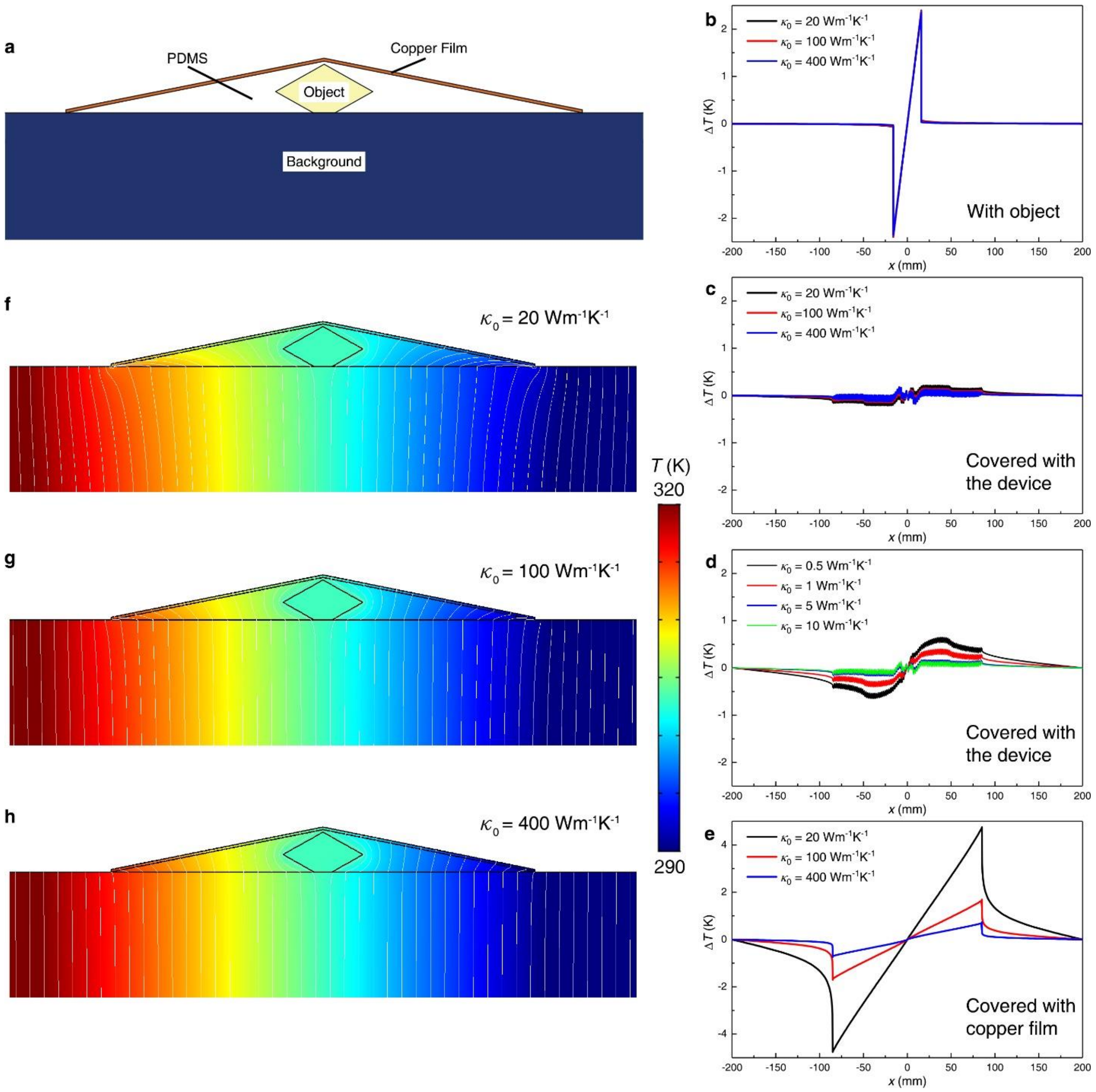

Supplementary Figure 2. Simulated results for different background materials. (**a**) Schematic graph of the strategy of covering a copper film (which does not work). (**b-d**) Temperature deviation from the theoretical solution for background material of different thermal conductivity κ_0, with (**b**) only an object on surface, (**c**,**d**) an object and covered by the device in main article, and (**e**) an object and covered by a copper film. (**f-h**) The temperature distributions for the strategy in (**a**) with different background materials. The black lines are boundaries for different materials. The white lines are isothermal lines.
